# Sevoflurane Inhibits the Proliferation of Neural Precursor Cells and Neural Migration of Mice by Inducing Iron Metabolism Disorders

**DOI:** 10.1111/cns.70369

**Published:** 2025-04-09

**Authors:** Xincheng Li, Runjiao Cheng, Mahammad Naeem, Xiaoou Nie, Jiaqi Wang, Liqiang Zhao, Xiaopeng Liu, Zhenhua Shi, Jianhua Zhang

**Affiliations:** ^1^ Laboratory of Molecular Iron Metabolism College of Life Science, Hebei Normal University Shijiazhuang Hebei Province China; ^2^ The Second Affiliated Hospital of Hebei Medical University Shijiazhuang Hebei Province China

**Keywords:** cell proliferation, cortical development, iron metabolism, neuronal migration, sevoflurane

## Abstract

**Background:**

Sevoflurane (Sev) is a volatile anesthetic and inhibits the proliferation of neural precursor cells (NPCs) and neuronal migration in the embryonic brain, thereby affecting offspring's cortical development and cognitive function.

**Methods:**

Pregnant mice were treated with 2.5% Sev. In utero, plasmids with GFP were electroporated into embryonic cortical neural precursor cells. Cell proliferation and neurite growth were detected by immunofluorescence of Ki67, pH 3, BrdU, Map2, and phalloidin labeling, respectively. Ferritin, transferrin receptor1 (TfR1), and confilin were detected by western blot.

**Results:**

Sev inhibited the proliferation of NPCs by down‐regulating the expression of pH 3 and Ki67, and also delayed the radial migration of cortical neurons. Sev impaired the multipolar‐to‐bipolar transition of migrating neurons by affecting Golgi orientation. Furthermore, Sev down‐regulated the expression of TfR1and increased the protein levels of ferritin heavy chain (FtH) and ferritin light chain (FtL) and caused the iron accumulation in the brain. Meanwhile, Sev induced the abnormal depolymerization and polymerization of microfilaments by increasing the ratio of p‐Cofilin/Cofilin and decreasing the ratio of F‐actin/G‐actin. Meanwhile, Sev inhibited cortical development by decreasing the neurite growth and number of branches of neurites. DFO, an iron‐chelating agent, could significantly ameliorate the inhibitory effect of Sev on the proliferation of NPCs and radial migration of projection neurons.

**Conclusions:**

Sev inhibited the NPCs proliferation and neuronal migration by inducing iron metabolic dysfunction. Regulating iron homeostasis could protect the cortical development of the embryo against Sev exposure during pregnancy.

## Graphical abstract

Sevoflurane inhibits the proliferation of neural precursor cells and neural migration of embryo brain in mice by inducing iron metabolism disorders. Maintaining iron homeostasis may be an effective strategy for treating Sev‐induced cortical dysplasia.

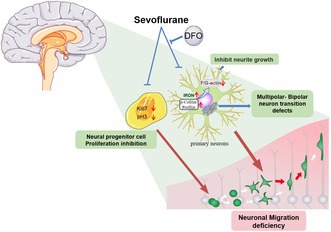

## 1 Introduction

Pregnancy is the critical period of fetal brain development, which is most vulnerable to environmental factors such as drugs, radiation, toxins, and anesthetics [1, 2, 3, 4]. The data from clinical studies show that 0.75%–4.8% of pregnant women undergo nonobstetric surgery for emergency conditions such as severe physical injuries and life‐threatening diseases [5]. However, the toxic effects of common anesthetics on fetal brain development and the mechanism through which inducing the serious complications are still unclear. In 2017, The US Food and Drug Administration (FDA) warned that repeated or prolonged use of anesthetics during surgery in pregnant women during the third trimester may negatively affect fetal brain development [6]. Sevoflurane (Sev) is the most used anesthetic during clinical surgery. Maternal Sev exposure during the first trimester of pregnancy can cause abnormal neuronal differentiation in fetal brain development [5] but also disrupts the oligodendrocyte myelination of the postnatal hippocampus and induces cognitive and motor impairments in offspring [7]. Iron (Fe) is a necessary cofactor of many metabolic processes in the central nervous system, including oxidative phosphorylation, DNA synthesis, neurotransmitter production, and oxygen transport. Therefore, the maintenance of iron homeostasis plays an important role in maintaining the normal physiological function of cells. Ferritin, including heavy chain ferritin (FtH) and light chain ferritin (FtL), is an iron storage protein, and transferrin receptor1 (TfR1) is responsible for getting iron into the cell. When cellular iron overloads, the expression of ferritin up‐regulates, and the excess iron binds to ferritin to eliminate the toxic effects of excess iron. Importantly, TfR1 expression decreases to keep cellular iron homeostasis because the excess iron can induce oxidative stress through the Fenton reaction, damaging the tissues. Thus, these proteins play an important role in maintaining the homeostasis of iron metabolism in the physiological process. Our previous work showed that Sev anesthesia induces cognitive impairment in mice during pregnancy by causing iron metabolism dysfunction and inhibiting myelinogenesis in the offspring [8]. All these findings suggest the effects of Sev on embryonic brain development. However, the underlying mechanism of maternal anesthesia on embryonic development and its neurotoxicity is not fully understood.

The second trimester is a critical period in the development of the embryonic tissues, which leads to neuronal migration and formation of the cortex tissues. The cortex in the mammalian brain is the most complex region coordinating the cognitive processes and hippocampus region [9]. Deficient or obstructed migration can lead to anencephaly, behavioral and mental defects. Alcohol exposure, ionizing radiation, and cytotoxic drugs can inhibit neuronal migration and cause neural dysfunction [10, 11]. Neuron migration is a complex process of cortical development. Recently, Chai et al. identified that Sev inhibits neuronal migration and axon growth in the developing mouse cerebral cortex by down‐regulating the cellular expression of neuro‐oncological ventral antigen 2 (Nova2) [12], suggesting that the effects of anesthetics on offspring cognitive function may appear on cortical development.

In this study, we hypothesize that Sev may induce abnormal migration of embryonic neurons by affecting iron metabolism disorders. Therefore, this study aimed to elucidate the mechanism of maternal anesthesia on embryonic development and neurotoxicity induced by Sev.

## 2 Methods

### 2.1 Animals

All C57BL/6 background mice used in this study were handled by the guidelines of the institutional animal care and committee of Hebei Normal University, China. Healthy 2‐month‐old Kunming female mice were obtained from the Laboratory of Animal Center of Hebei Medical University. Female mice were maintained on a 12‐h light/dark cycle and were bred overnight with C57BL/6N males. Healthy C57BL/6N male mice aged 2 months, purchased from Vitong Lihua, Beijing. The offspring of Kunming female mice were mated with C57BL/6N male mice and recognized as the F1 generation. F1 female mice were bred overnight with C57BL/6N male mice. All mice used in this experiment were aged 2–6 months.

### 2.2 Animal Anesthesia Treatment

Embryonic stage 15.5 days (E15.5) female mice of the F1 generation were randomly divided into control and Sev‐treated groups. The Sev group was subjected to continuous anesthesia with 2.5% Sev + 40% O_2_ for 2 h for two consecutive days. Some of the pregnant mice used for neuronal migration analysis were injected with BrdU (20 mg/mL and 50 mg/kg) through the tail vein for 3 h before Sev treatment, and the embryonic mice were killed at E18.5 for follow‐up experiments.

### 2.3 BrdU and Desferrioxamine (DFO) Treatment

Briefly, when using BrdU incorporation assays to monitor cell proliferation, BrdU (50 mg/kg) was injected into pregnant mice through the tail vein 2 h before tissue collection at E18.5. Anesthesia was administered between 15:00 and 17:00. During anesthesia, the female mice were placed on a heating pad at 37℃ to maintain body temperature. Some of the pregnant mice were injected with BrdU and DFO (an iron chelating agent) mixed solution by intraperitoneal administration 3 h before Sev treatment with a BrdU dose of 50 mg/kg (concentration of 20 mg/mL) and DFO amount of 25 mg/kg with a concentration of 10 mg/mL. As for ferric ammonium citrate (FAC) group, We performed intra‐cerebroventricular injection (i.c.v.) of FAC (1μg/μl) 1μl in embryonic mice at E15.5. Embryonic mice were killed at E18.5 for follow‐up experiments.

### 2.4 In Utero Electroporation

Sev‐treatment was performed after in utero electroporation of the embryos. In utero electroporation was performed at E14.5 or E15.5 through the addition of the anesthetic agent, 2,2,2‐Tribromoethanol, and Sev‐treatment at E15.5 and E16.5, respectively.

In utero electroporation was carried out as previously described [13]. Pregnant mice were anesthetized, and their uterine horns were exposed with a midline laparotomy incision. Embryos were removed and carefully placed on humidified gauze pads. Plasmid DNA and 0.01% fast green (Fluka) were injected into the embryonic brain's lateral ventricles by a glass micropipette. For electroporation, 5 × 50 ms, 37 V square pulses separated by 950 ms intervals were delivered with forceps‐type electrodes connected to an ECM 830 electroporator (BTX Harvard Apparatus). The uterus was then replaced into the abdominal cavity, and the abdomen wall and skin were sutured using a surgical needle and thread. The pregnant mice were warmed in an incubator until they regained consciousness, and embryos were allowed to develop in utero for the time indicated.

### 2.5 Primary Cortical Neuronal and Neuro‐2a (N2a) Cell Cultures and Sev Treatment

Primary cortical neurons were isolated and cultured as described previously [14]. Neuro‐2a cells were stored in our laboratory. Briefly, embryos (E16.5) were removed from anesthetized pregnant F1 female mice. Cerebral cortices were dissected out and chopped into small pieces after the meninges were removed thoroughly. After incubation in HBSS solution containing 0.125% (w/v) trypsin for 20 min at 37℃, digested tissues were mechanically triturated by repeated passages through a Pasteur pipette in PBS solution containing 0.05% (w/v) DNase. Dissociated cells were suspended in the neurobasal medium with B‐27 supplement, and 100 U/mL penicillin/streptomycin and plated on poly‐D‐lysine coated dishes. Incubation was conducted at 37℃ in a 5% CO_2_ atmosphere. Primary cells and N2a cells were cultured for 24 h and then treated with 5% Sev for 6 h at 37℃ in a 5% CO_2_. From the beginning of primary cell isolation, the cells were cultured in vitro for 4 days and 7 days, respectively, and then labeled with phalloidin (Cat. No. YP0052S, US Everbright, Shanghai, China) and Map2 (Cat. No. M1406, Sigma, St. Louis, MO).

### 2.6 Immunofluorescent Staining

The immunohistochemistry assay was performed as in our previously described study [8]. Briefly, the brain sections of the embryonic mice were placed in a 65°C oven for 40–60 min and washed with 0.01 M PBS for three times. Then, slices were placed into 0.01 M citrate buffer and boiled for 10 min. The sections were soaked in 0.01 M PBS (1% TritonX‐100 and 0.5% Tween‐20) at room temperature for 7 min. The slices were washed three times with 0.01 M PBS for 5 min each time. The samples were incubated overnight at 4°C with the primary antibodies of anti‐pH 3 antibody (Cat. No. 9706, Cell Signaling Technology, MA, USA), anti‐ferritin (Cat. No. ab75973, Abcam, Cambridge, UK), anti‐Ki67 (Cat. No. ab15580, Abcam, Cambridge, UK), anti‐BrdU (Cat. No. ab6326, Abcam, Cambridge, UK), anti‐GFP (Cat. No. ab13970, Abcam, Cambridge, UK), anti‐GM130 (Cat. No. 610823, BD Biosciences, San Jose, CA, USA). Rhodamine‐conjugated and FITC‐conjugated secondary antibodies were used. Images were captured using a ZEISS LSM710 (LSM710; ZEISS, Germany).

### 2.7 Western Blot Analysis

The samples of cortex regions were placed into RIPA lysate and centrifuged at 12000 g for 20 min. The supernatant holding the proteins was estimated by a protein quantification analysis kit (KangWei, Beijing, China). The western blot analysis was performed as previously described [8]. The following antibodies were used: Profilin1 (Cat. No. ab50667, Abcam, Cambridge, UK), Cofilin (Cat. No. 66057‐1‐lg, Proteintech, Wuhan, China), p‐Cofilin (Cat. No. 3313, Cell Signaling Technology, MA, USA), TfR1 (Cat. No. 13‐6890, ThermoFisher, USA), FtL (Cat. No. ab109373, Abcam, Cambridge, UK), FtH (Cat. No. ab183781, Abcam, Cambridge, UK), β‐actin (Cat. No. CW0096M, KangWei, Beijing, China), GAPDH (Cat. No. 60004‐1‐Ig, Proteintech, Wuhan, China).

### 2.8 Microscope Image Acquisition

Brain tissues through the somatosensory cortex were observed through laser scanning confocal microscopy (ZEISS LSM900), and only the brightness, contrast, and color balance were optimized after acquisition. Cortical subregions were identified based on cell density by using DAPI staining. The numbers of disorientated, multipolar, and bipolar cells were counted in the IZ with ImageJ software. Misorientated cells were defined as cells with a leading process oriented at an angle of 30° off the normal pial surface‐directed radial direction (measured with ImageJ software); multipolar cells were defined as cells with more than three processes.

### 2.9 Statistical Analysis

In this study, GraphPad Prism 7 (GraphPad Software, USA) was used to analyze the data as mean ± SEM. All western blotting data were obtained based on gray values using Image J Software, and the ratio of target protein gray value to actin gray value in the control group was 1. The normality of the data was analyzed using the Shapiro–Wilk test. For data with normal distribution, comparisons were performed using the unpaired *t*‐test or multiple *t*‐tests. Continuous variables data with nonnormal distributions were analyzed by the Mann–Whitney test. All data are expressed as mean ± SEM. Three to four embryonic mice per group were used to test the relative protein expression by western blot, and three mice per group were used for immunostaining studies, respectively. In the test of the proliferation of neural precursor cells and neuronal migration, one‐way ANOVA were used to analyze the differences among the control group, Sev treatment group, DFO treatment group, and Sev + DFO treatment group. The difference in the two‐group comparison was determined by using a Student's *t*‐test when the data passed normality testing. One‐way ANOVA and individual Student's *t*‐test with Bonferroni correction were used to determine differences among groups in profilin 1, confilin, G‐actin, F‐actin, FtH, FtL, and TfR1. A probability level of 95% (*p* < 0.05) was considered statistically significant, and significance testing was two‐tailed.

## 3 Results

### 3.1 Sev Exposure Inhibited Proliferation of Neural Precursor Cells (NPCs) and Neuronal Migration in Embryonic Mice

**FIGURE 1:**
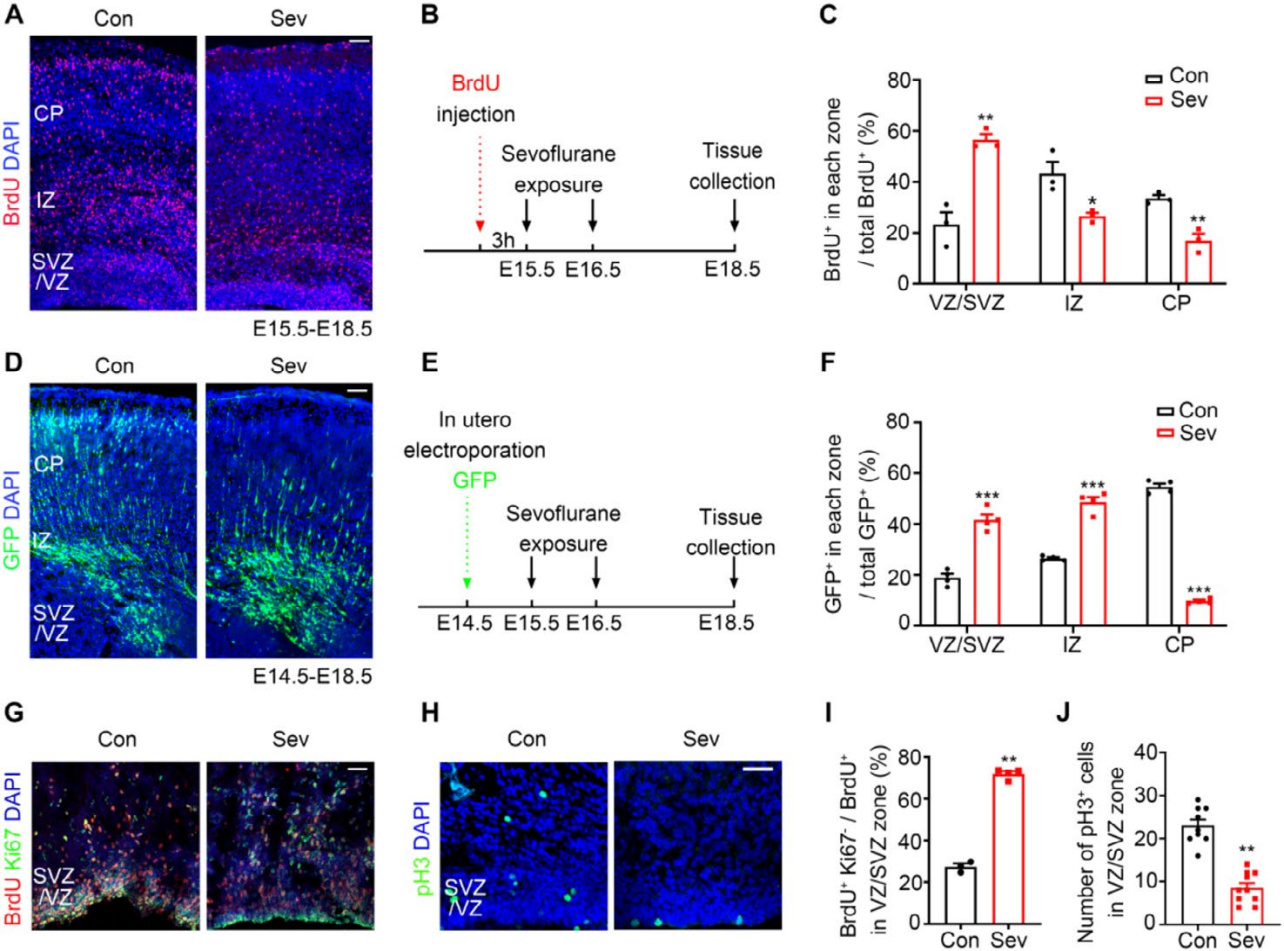
Sev treatment leads to neuronal migration defects and inhibits NPCs proliferation in embryonic mice. (A) Pregnant mice were administered BrdU at E15.5 and followed by control or Sev treatment. Coronal sections of each brain were collected at E18.5 and immunostained for BrdU and DAPI. The neocortex is divided into the VZ/SVZ, IZ, and CP based on DAPI staining. (B) Experimental design of BrdU‐labeled cells and Sev treatment. (C) The statistical diagram of Figure 1A (the percentage of BrdU‐positive cells in VZ/SVZ, IZ, and CP regions accounted for the total BrdU‐positive cells). (D) Neurons were electroporated with GFP plasmid and the migration process was evaluated by analyzing the distribution of GFP‐positive neurons. (E) Experimental design of GFP‐labeled neuron cells and Sev treatment. (F) The statistical diagram of Figure 1D (the percentage of GFP‐positive cells in VZ/SVZ, IZ, and CP regions accounted for the total GFP‐positive cells). (G) E15.5 embryos were treated with control or Sev, and E18.5 brains with a 2 h BrdU labeling were co‐immunostained for BrdU (red) and Ki67 (green). (H) Immunostaining of pH 3 of E18.5 control and Sev treated cortical slices. (I, J) The statistical diagram of (G) and (H). All the data are expressed as average ± SEM. *n* = 3‐10, *p < 0.05 versus control group, **p < 0.01 versus control group, ***p < 0.001 versus control group. Scale bars, 50 μm.

In the developing neocortex, NPCs in the ventricular/subventricular zone (VZ/SVZ) region proliferate and differentiate to form new NPCs and neurons, while neurons in the intermediate zone (IZ) and cortical plate (CP) regions do not proliferate. To evaluate the effect of Sev on neuronal migration in embryonic mice, we used BrdU to label the proliferating NPCs and proliferated NPCs in the embryonic mouse brain and counted the proportion of each layer in the VZ/SVZ‐IZ‐CP migration path. Our results showed that the number of BrdU‐positive cells in the VZ/SVZ of the Sev‐treated group accounted for 60% of the whole cortical area. However, the number of BrdU‐positive cells in the control group accounted for only 20%, suggesting that most cells were arrested in the VZ/SVZ (*t*_4_ = 6.236, *p* = 0.0033, *n* = 3). The proportion of BrdU‐positive cells in the IZ zone was decreased (*t*_4_ = 3.426, *p* = 0.0266, *n* = 3), and BrdU‐positive cells entering the CP zone also reduced significantly (*t*_4_ = 5.561, *p* = 0.0051, *n* = 3) at E18.5 as shown in Figure 1A–C, indicating that Sev exposure inhibited neuronal migration.

Radial migration is a crucial process for the developing neocortex in the mammalian brain, which is closely related to the proliferation and differentiation of NPCs. To further clarify the effect of Sev on neuronal migration, we used electroporated GFP plasmids to label the neurons in utero electroporation at E14.5. Then, the distribution of GFP‐positive neurons in the brain sections at E18.5 was used to verify the neuronal migration defects. Similar to the results of BrdU labeling, GFP‐positive cells in Sev‐treated group were primarily accumulated in VZ/SVZ (*t_6_ =8.433, P = 0.0001, n=4*) and IZ regions *(t_6_ =10.84, P < 0.0001, n=4)*, and the number of cells entering the CP region (*t_6_ = 32.02, P < 0.0001, n=4)* was significantly less than that in the control group as shown in Figure 1D–F, indicating that Sev treatment resulted in neuronal migration defects.

Sev treatment increased the accumulation of newborn cells in the VZ/SVZ, and any defects occurring during NPCs' proliferation may also lead to impaired neuronal migration. Therefore, we investigated whether Sev treatment affected the proliferation of NPCs. Ki67 is an important marker of cell proliferation, and phosphorylated histone H3 (pH 3) is a marker that indicates cells are undergoing mitosis. Mitotic cells in the VZ/SVZ of E18.5 brain slices were identified by immunostaining to analyze the mitotic index. The results showed that the percentage of BrdU^+^Ki67^−^ cells among total BrdU^+^ cells (about 80%) was increased significantly in the VZ/SVZ region compared with that of the control group (about 28%) after Sev treatment (Figure 1G,I), indicating that the number of NPCs exiting the cell cycle increased. The number of pH 3^+^ cells in the Sev‐treated group was significantly decreased (Figure 1H,J) (t_17_ =8.093, P < 0.001, n=9‐10), suggesting that Sev decreased the number of NPCs in mitosis. Our results showed that the cell proliferation process regulated by Ki67 and pH 3 may be related to the cell arrest process induced by Sev.

### 3.2 Sev Impaired the Multipolar to Bipolar Transition of Migrating Neurons

**FIGURE 2:**
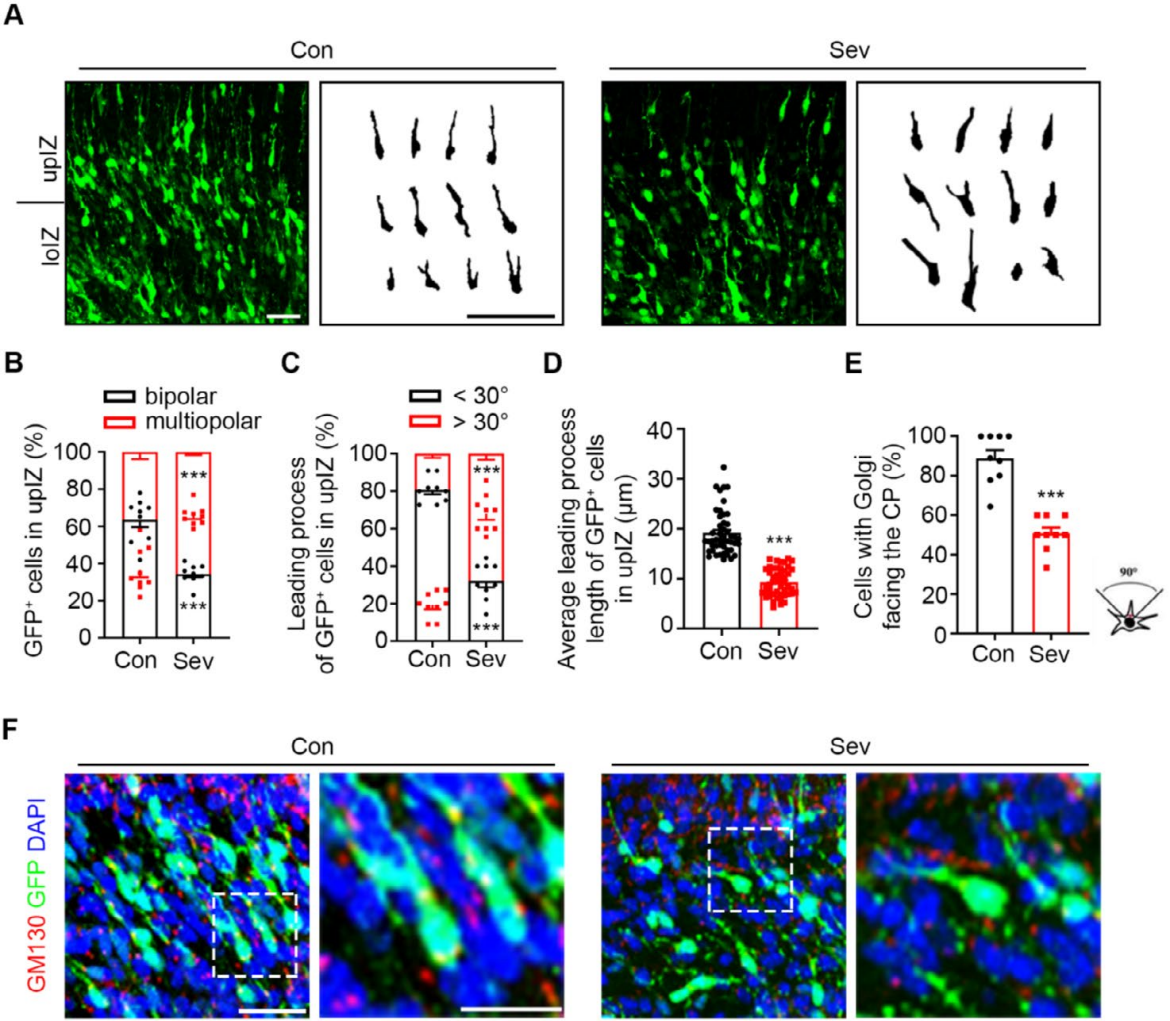
Sev treatment results in an abnormal multipolar‐to‐bipolar transition of migrating neurons. (A) Representative images of GFP‐transfected neurons in the loIZ and upIZ in control and Sev‐treated E18.5 cortex. (B) The statistical analysis of the proportion of multipolar and bipolar neurons in the upIZ region of E18.5 mouse cortices. (C) The statistical analysis of the direction of the leading process in the upIZ region. (D) The statistical analysis of the average length of the leading process in the IZ region. (E) Percentages of cells with Golgi facing the CP in the IZ. (F) The Golgi was labeled with anti‐GM130 antibody. Sev induced an abnormal orientation of the neuronal Golgi apparatus. All the data are expressed as average ± SEM. *n* = 9‐48, ***p < 0.001 versus control group. Scale bars, 50 μm.

Early immature neurons located in SVZ and lower IZ (loIZ) showed a multipolar morphology with multiple neurites, revealing random migration of neurons. After completing the multipolar to bipolar transition in the upper IZ (upIZ), the neurons migrated along the radial glial fibers toward the CP. We analyzed the morphology of GFP^+^ neurons to evaluate whether Sev treatment affected the morphological transition process. Morphological analysis revealed that most of the GFP^+^ control neurons had a leading process pointing toward the CP, indicating that they have completed the morphological transition from multipolar to bipolar. However, in the Sev‐treated group, more GFP^+^ neurons had multiple shorter processes and exhibited a multipolar morphology (Figure 2A). The number of GFP^+^ neurons converted to bipolar morphology decreased significantly (t_16_ =6.765, P < 0.0001, n=9) (Figure 2B), suggesting a defect in the multipolar‐to‐bipolar transition of these neurons. In addition, these neurons usually displayed abnormal orientation angle values (t_16_=12.13, P < 0.0001, n=9) (Figure 2C) and shorter leading processes (U=3, P < 0.0001, n=48)   (Figure 2D). These results suggested that Sev treatment disrupted the multipolar to bipolar transition and consequently impaired neuronal migration. The establishment of cell polarity, in which the orientation of the Golgi apparatus can be detected, is a critical phenomenon in the morphological transformation of neurons. We therefore used a Golgi marker, GM130, to analyze the orientation of the Golgi apparatus in the loIZ neurons. Fewer loIZ neurons with correct Golgi orientation in Sev‐treated cortical slices compared with the control group (Figure 2E,F) (t_16_ =7.349, P < 0.0001, n=9), indicating that Sev treatment affected Golgi orientation during the morphological transformation of neurons.

### 3.3 Sev Disrupted the Dynamics of the Actin Cytoskeleton

**FIGURE 3:**
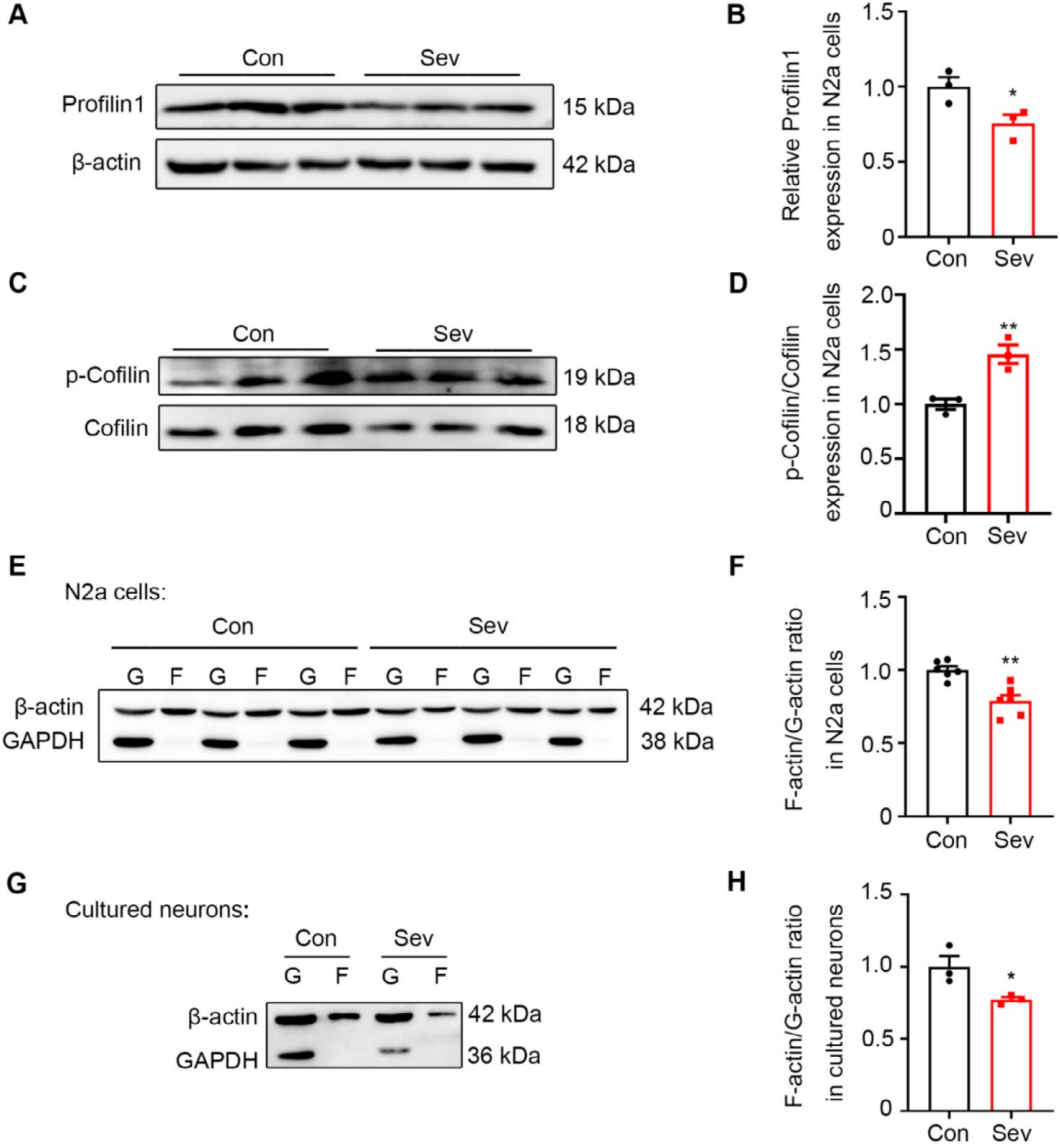
The effect of Sev on the levels of Actin‐binding proteins profilin 1, phosphorylation of cofilin, and F‐Actin/G‐Actin ratio. (A, B) Sev significantly decreased the expression of profilin 1 in N2a cells. (C, D) Sev significantly increased the ratio of p‐Cofilin/Cofilin in N2a cells. (E, F) Sev significantly decreased the F‐Actin/G‐Actin ratio in N2a cells. (G, H) Sev significantly decreased the ratio of F‐Actin/G‐Actin in the cultured primary cortical neurons isolated from E16.5 pups. All the data are expressed as average ± SEM. *n* = 3‐6, *p < 0.05 versus control group, **p < 0.01 versus control group.

The neuronal migration depends on continuous actin cytoskeleton [15]. Profilin 1 is an actin‐binding protein that regulates the actin polymerization in cells. In addition, cofilin, an actin‐depolymerizing factor, is a major regulator of actin dynamics involved in regulating cell migration, and phosphorylation at Ser‐3 inactivates the actin‐depolymerizing activity of cofilin. To assess whether Sev inhibited neuronal migration by profilin 1 and cofilin, we detected the levels and activities of profilin 1 and cofilin via Western blot. Sev treatment significantly decreased the protein level of profilin 1 (*t*_4_ = 2.875, *p* = 0.0452, *n* = 3) (Figure 3A,B), and increased the phosphorylation of cofilin (p‐Cofilin) (*t*_16_ = 3.112, *p* = 0.0067, *n* = 3) (Figure 3C,D), indicating that Sev treatment affected the polymerization and depolymerization of microfilament. F‐actin and G‐actin represent the polymerization and monomer morphology of actin, respectively; therefore, we performed protein fractionation of F‐actin and G‐actin to test whether the F/G‐actin ratio was altered in Sev‐treated Neuro‐2a cells. A noticeable decrease in F‐actin/G‐actin was observed in Sev‐treated cells compared with that of the control group (*t*_10_ = 4.410, *p* = 0.0013, *n* = 6) (Figure 3E,F). To verify if this effect also occurred in Sev treated primary neurons of embryonic cortex, we further tested the level of F‐actin and G‐actin in the primary cortical neurons which were isolated from E16.5 pups. The results showed that the ratio of F‐actin/G‐actin was decreased significantly (*t*_4_ = 2.972, *p* = 0.0411, *n* = 3) (Figure 3G,H). These data indicated that Sev treatment disrupted the actin dynamics.

### 3.4 Sev‐Induced Iron Accumulation in the Cortex

**FIGURE 4:**
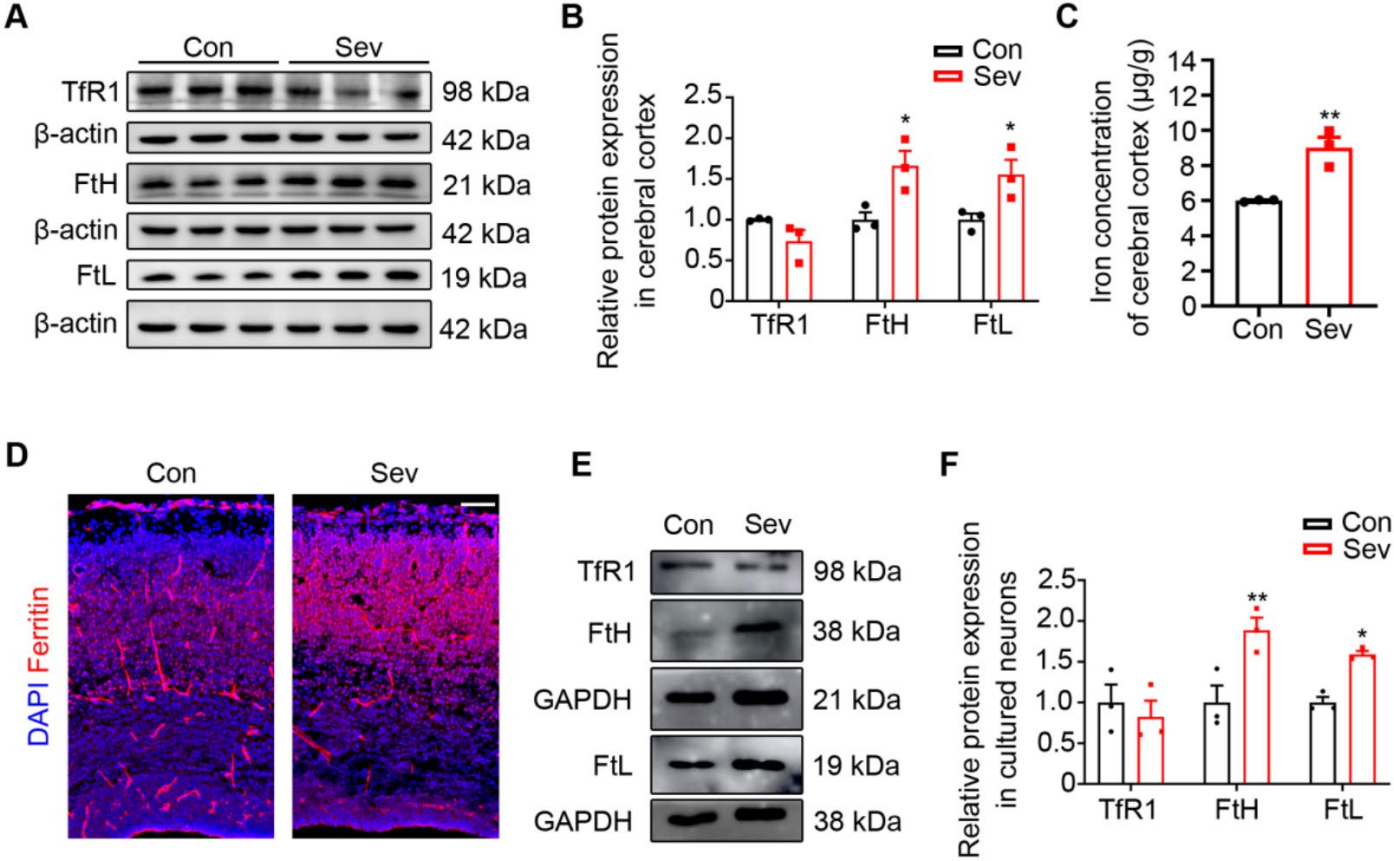
Sev induced increased iron levels in the embryonic neocortex. (A, B). Sev decreased the expression of TfR1 and increased the FtH and FtL in the embryonic cerebral cortex. (C) ICP‐MS showed the iron levels in the embryonic cerebral cortex. (D) The expression of ferritin in the cortex was assayed by immunofluorescence. (E, F) Sev decreased the expression of TfR1 and increased the FtH and FtL in primary cortical neurons of embryo brain. All the data are expressed as average ± SEM. *n* = 3, *p < 0.05 versus control group, **p < 0.01 versus control group.

Iron is one of the essential trace elements for body metabolism. To assess whether Sev affected neuronal migration by disrupting iron metabolism, the expression levels of iron metabolism‐related proteins FtH, FtL, and TfR1 were examined in the cortex of E18.5 embryonic mice. Our results showed that Sev down‐regulated the expression of TfR1 (*t*_4_ = 1.945, *p* = 0.12, *n* = 3), but significantly increased the protein levels of FtH (*t*_4_ = 3.270, *p* = 0.0307, *n* = 3) and FtL (*t*_4_ = 2.818, *p* = 0.0479, *n* = 3) (Figure 4A,B), indicating that Sev induced the iron accumulation in cortex, which was in accordance with the iron concentration (*t*_4_ = 5.070, *p* = 0.0071, *n* = 3) detected by ICP‐MS, as shown in Figure 4C. We further performed immunostaining for ferritin to evaluate the expression of ferritin. We found that the fluorescence intensity of ferritin in the Sev group was much stronger than that in the control group, as shown in Figure 4D, suggesting that Sev induced the iron overload in the cortex. In addition, using in vitro cultured primary cortical neurons, we found that Sev treated for 6 h showed an increase in protein levels of FtH (*t*_4_ = 3.409, *p* = 0.0270, *n* = 3) and FtL (*t*_4_ = 7.307, *p* = 0.0018, *n* = 3) and had no significant effect on TfR1 (*t*_4_ = 0.5939, *p* = 0.5845, *n* = 3) (Figure 4E,F). These data suggest that Sev‐induced iron accumulation may be a critical factor affecting neuronal migration.

### 3.5 Sev Inhibited the Development of Neurites

**FIGURE 5:**
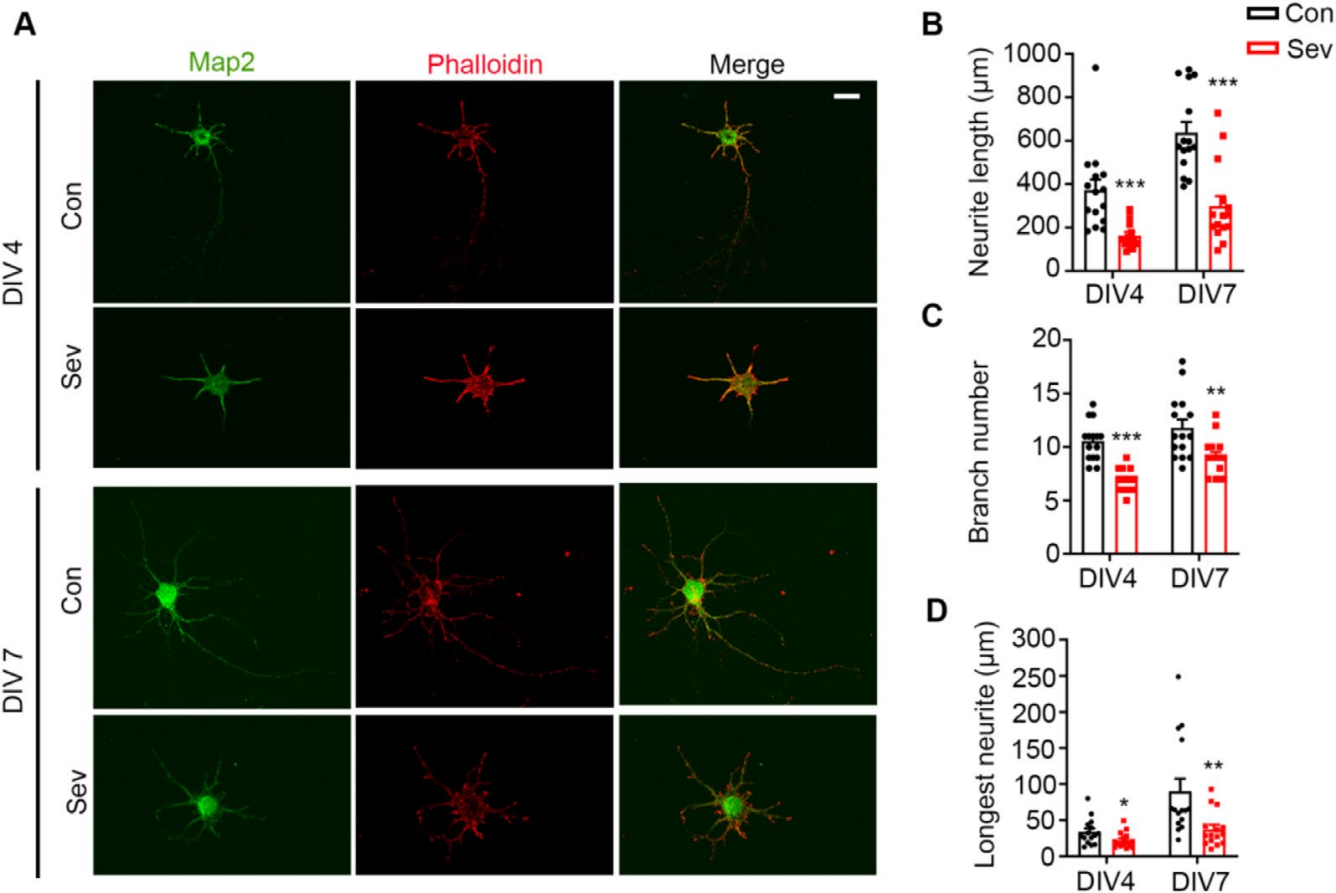
Sev inhibited the development of neurites. (A) Primary cortical neurons were labeled with phalloidin and Map2 to detect the branches and lengths of neurites. (B–D) Statistical analysis of neurite length, number of neurite branches, and the length of the longest neurite. All the data are expressed as average ± SEM. *n* = 15, *p < 0.05 versus control group, **p < 0.01 versus control group, ***p < 0.01 versus control group. Scale bar, 50 μm.

The formation of microfilaments is closely related to neurite growth, which can reflect the development of neurons. To detect the effect of Sev on neurite growth during development, we labeled microfilaments in primary neurons of embryonic mice with phalloidin and Map2, and measured the length and branches of neurites at days in vitro (DIV) 4 and DIV 7 after treatment with Sev. We found that Sev decreased the total neurite length (DIV4: *t*_28_ = 4.075, *p* = 0.003, *n* = 15, DIV7: *t*_4_ = 4.987, *p* < 0.001, *n* = 15), branch number (DIV4: *t*_28_ = 6.777, *p* < 0.001, *n* = 15, DIV7: *t*_28_ = 3.044, *p* = 0.0050, *n* = 15) and the longest neurite length (DIV4: *t*_28_ = 2.133, *p* = 0.0419, *n* = 15, DIV7: *t*_28_ = 2.846, *p* = 0.0081, *n* = 15) (Figure 5A–D), which indicating that Sev inhibited neurite development.

### 3.6 Iron Overload Suppressed Neuronal Migration and Cell Proliferation

**FIGURE 6:**
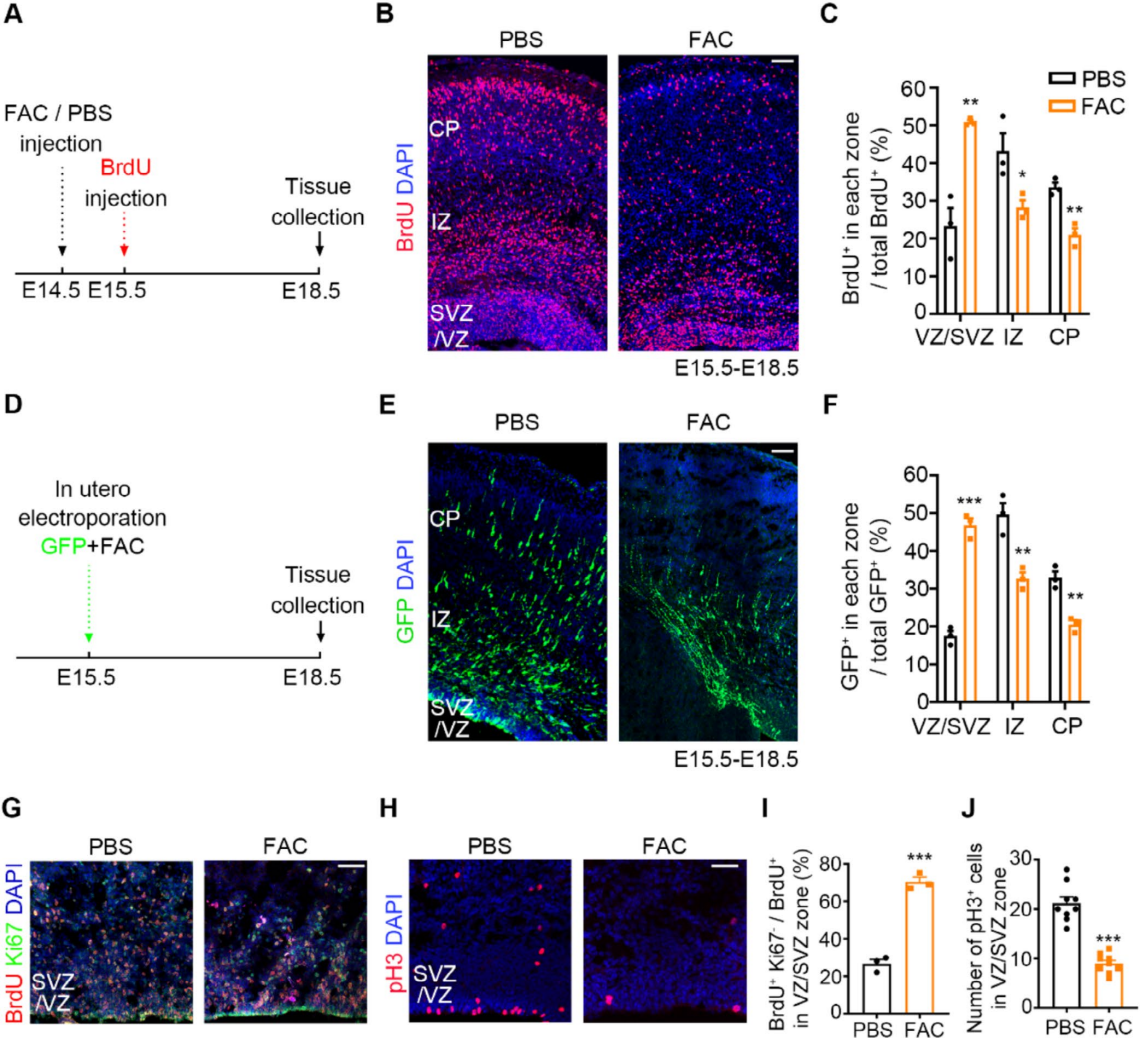
Iron overload suppressed the neuronal migration and cell proliferation. (A) Experimental design of PBS/FAC treated and BrdU injection mice. (B, C) FAC inhibited the migration of BrdU^+^ cells from the VZ/SVZ to the IZ and CP region. (D) Experimental design of mice treated with FAC after electroporated cells with GFP plasmid. (E, F) FAC inhibited the migration of GFP^+^ cells from the VZ/SVZ to the IZ and CP region. (G) E15.5 embryos were injected with PBS or FAC, and E18.5 brains with a 2 h BrdU labeling were co‐immunostained for BrdU (red) and Ki67 (green). (H) Immunostaining of pH 3 of E18.5 PBS and FAC injected cortical slices. (I, J) The statistical diagram of (G) and (H). FAC inhibited cell proliferation in VZ/SVZ. All the data are expressed as average ± SEM. *n* = 3‐9, * *p* < 0.05 versus control group, **p < 0.01 versus control group, ***p < 0.001 versus control group. Scale bars, 50 μm.

To further determine whether the abnormal neuronal migration caused by Sev treatment is associated with the increase of cortical iron level, we injected FAC (1 μg/μL) into the lateral ventricle of E14.5 embryonic mice to increase the brain iron level and then labeled newly generated cells with BrdU at E15.5, and the distribution of BrdU^+^ cells in the neocortex was analyzed at E18.5. We found that elevated cortical iron levels decreased the number of BrdU^+^ cells in the IZ (*t*_4_ = 2.961, *p* = 0.0415, *n* = 3) and CP (*t*_4_ = 5.757, *p* = 0.0451, *n* = 3), but increased the number of BrdU^+^ cells in the VZ/SVZ (*t*_4_ = 5.700, *p* = 0.0046, *n* = 3) (Figure 6A–C). To further confirm these results, we electroporated E15.5 brains with GFP plasmid to label newly generated cells and simultaneously injected FAC into the lateral ventricles. The results were consistent with the findings in the BrdU‐labeled experiment, as shown in Figure 6D–F, there was an accumulation of GFP^+^ cells in the VZ/SVZ (*t*_4_ = 12.91, *p* = 0.0002, *n* = 3) and a decrease in GFP^+^ cells in the IZ (*t*_4_ = 4.900, *p* = 0.0080, *n* = 3) and CP (*t*_4_ = 6.017, *p* = 0.0038, *n* = 3) at E18.5. These data suggest that the elevation of cortical iron level caused by Sev inhibits the radial neuronal migration.

To verify the effect of elevated iron on cell proliferation, BrdU labeling was performed at E18.5 and brain slices were analyzed at 2 h post‐BrdU injection. We co‐stained for BrdU and Ki67 to reveal the cells that withdrew from the cell cycle (BrdU^+^ and Ki67^−^) in the VZ/SVZ at E18.5 (Figure 6G). The percentage of BrdU^+^; Ki67^−^ cells among total BrdU^+^ cells was increased in the FAC treatment group, suggesting that more NPCs had exited the cell cycle after FAC injection (*t*_4_ = 12.71, *p* = 0.0002, *n* = 3) (Figure 6I). In addition, E18.5 brain slices were immunostained with anti‐pH 3 antibody to analyze the mitotic index (Figure 6H). Quantitative analysis showed that the number of pH 3^+^ cells in the VZ and SVZ was significantly decreased in FAC injection brains compared to that of controls (*t*_16_ = 8.596, *p* < 0.0001, *n* = 9) (Figure 6J). The results indicated that elevated iron could inhibit cell proliferation.

### 3.7 DFO Treatment Alleviated Sev‐Induced Iron Elevation and Rescued Migration and Proliferation Defects in the Cortex

**FIGURE 7:**
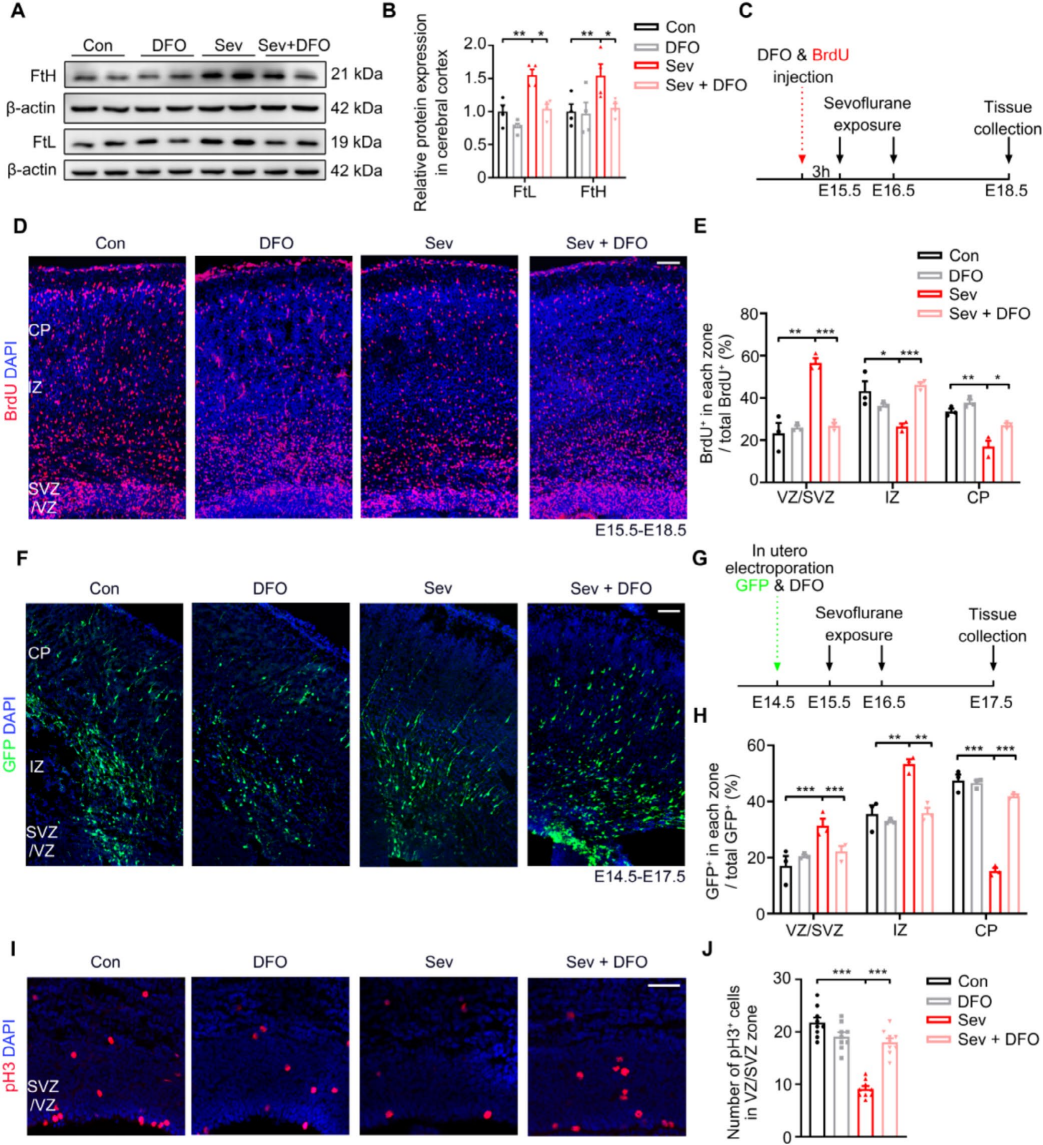
DFO pretreatment alleviates the elevated iron level and abnormal proliferation and migration in the cortex of embryonic mice caused by Sev. (A, B) DFO inhibited the increase of FtH and FtL expression induced by Sev. (C) Experimental design of mice injected with DFO and BrdU and followed by control or Sev treatment. (D, E) DFO alleviated Sev‐induced neuronal migration defects by analyzing the distribution of BrdU^+^ cells in the neocortex. (F) Mouse embryos were electroporated with GFP plasmids and injected with DFO at E14.5, and brain slices were examined at E18.5 using an anti‐GFP antibody. (G) Experimental design of mice injected with DFO and electroporated with GFP and followed by control or Sev treatment. (H) Quantification of GFP^+^ neurons in the images shown in (F). DFO alleviated Sev‐induced neuronal migration defects by GFP fluorescence distribution. (I, J) Immunostaining of pH 3 of E18.5 cortical slices and analysis of the pH 3^+^ cell in the VZ/SVZ. DFO alleviated cell proliferation obstruction induced by Sev. All the data are expressed as average ± SEM. *n* = 3‐9, * *p* < 0.05, **p < 0.01, ***p < 0.001. Scale bars, 50 μm.

Increased iron levels may be one reason for the abnormal migration caused by Sev treatment, we hypothesized that alleviating the elevated iron level using DFO may partially rescue the migration defects in Sev‐treated mice. We first injected DFO (25 mg/kg) into the E15.5 mice through the tail vein and followed by with or without Sev treatment. At E18.5, we analyzedthe Ferritin (including FtH and FtL) expression in the neocortex. The results showed that DFO inhibited the increase of ferritin induced by Sev (FtH: *t*_6_ = 4.493, *p* = 0.0041, *n* = 4, FtL: *t*_6_ = 2.580, *p* = 0.0417, *n* = 4) (Figure 7A,B), indicating that DFO decreased the iron levels in the cortex. To determine whether DFO could rescue the migration defects in Sev‐treated mice, BrdU and DFO were injected into E15.5 mice by intraperitoneal administration with doses of 50 mg/kg and 25 mg/kg, respectively, followed by Sev treatment 3 h later. At E18.5, we analyzed the distribution of BrdU^+^ cells in the Sev + DFO group in the neocortex (Figure 7C,D). The proportion of BrdU^+^ cells increased in the CP (t_4_ =3.540, P = 0.0240, n=3)   and decreased in the VZ/SVZ (t_4_ =11.14, P = 0.0003, n=3) and IZ (t_4_ =10.55, P = 0.0004, n=3) significantly compared to the Sev treatment group (Figure 7E), which indicated that DFO could rescue the migration defects caused by Sev treatment. To strengthen these results, we also electroporated E15.5 brains with GFP plasmid and simultaneously treated with DFO, and analyzed the distribution of GFP^+^ cells in the neocortex at E18.5 (Figure 7G). Notably, the results showed that the distribution of GFP^+^ cells in the Sev + DFO group was similar to that in the control group (VZ/SVZ: *t*_4_ = 2.928, *p* = 0.0428, *n* = 3, IZ: *t*_4_ = 6.925, *p* = 0.0022, *n* = 3, CP: *t*_4_ = 21.45, *p* < 0.0001, *n* = 3) (Figure 7F,H), indicating that DFO indeed could rescue the migration defects in Sev‐treated mice. In addition, we observed that DFO treatment also increased pH 3^+^ Cells and decreased the Ki67^−^ cells, indicating that DFO restored the proliferation defect induced by Sev in NPCs (t_32_ =7.695, P < 0.0001, n=9) (Figure 7I,J). Taken together, these findings demonstrate that DFO preconditioning can alleviate the abnormal iron deposition and rescue the proliferation and migration defects in the embryonic cerebral cortex caused by Sev.

## 4 Discussion

Sev is the anesthetic agent used commonly in clinical surgeries. However, its safety has been established; recent studies in animals have shown that maternal exposure to Sev during pregnancy can affect the brain development of the offspring, resulting in cognitive impairment [5, 16]. Prenatal Sev exposure impairs the learning and memory of rat offspring through inflammasome activation [17]. Maternal Sev exposure also disrupts oligodendrocyte myelination of the postnatal hippocampus and induces cognitive and motor impairments in offspring. These data suggest that Sev may have affected the development of the embryonic mouse brain [7].

However, the mechanism by which Sev affects fetal brain development is not fully understood. The cortex is an important region of the brain for cognitive function. During cerebral cortex development, neural precursor cells proliferate and differentiate to form new neurons [18]. Neurons in the lower part of SVZ and IZ are in a multipolar state [19]. After stimulation by intracellular and extracellular signals, they complete the morphological transformation in the upper IZ region to form a bipolar state with an obvious leading process and migrate along radial glial fibers [20]. When their leading process attaches to the pial surface, the migration mode switches to stomal translocation to arrive at their final positions [21]. After that, neurons further initiate the development of dendrites and axons [22]. Therefore, neuron migration is a critical process in cortical development.

In this study, we evaluated whether Sev inhibits neuronal migration and cortical development in embryonic mice and explored a possible mechanism. E15.5 female mice is a critical period for the formation and development of the cerebral cortex of embryonic mice. After labeling NPCs with BrdU and GFP, we evaluated neuronal migration by examining neuronal distribution. We found that after E15.5 female mice were treated with 2.5% Sev for 2 h for two consecutive days, cortical neuronal migration was indeed inhibited in embryonic mice. Many neurons were delayed in the VZ/SVZ and IZ. The number of neurons decreased significantly in the CP. During cortical development, the migration of neurons from the VZ/SVZ to IZ and CP regions was a key process in cortical development. Our results showed that maternal Sev exposure inhibited neuronal migration in embryonic mice.

Neuronal migration depended on the proliferation of NPCs. Mature neurons were derived from the proliferation and differentiation of NPCs. Using BrdU, Ki67, and pH 3 antibody to label proliferating cells, we found that Sev‐treatment also inhibited the proliferation of NPCs in the VZ/SVZ. The proliferation of NPCs mainly occurred in the VZ/SVZ region, and postmitotic projection neurons derived from the VZ/SVZ migrate radially through the IZ to the CP of the developing neocortex. These data suggested that the inhibitory effect of Sev on NPCs proliferation might be one of the factors affecting neuron migration.

In the loIZ, neurons are in a multipolar state, and only after completing the morphological transformation in the upIZ region to form a bipolar state with an obvious leading process can they begin to move and migrate [23]. To assess whether Sev inhibited neuronal migration by affecting morphological transformation, we investigated the effect of Sev on the polarity transition of nerve cells, which played a key role in neuronal migration. We found that Sev causes some neurons to be delayed in the IZ of the cortex, a critical region for neurons to transform from multipolar to bipolar morphology [23]. To further clarify whether Sev inhibits radial migration by affecting neuronal morphological change, we used in utero electroporation to investigate further. Our results showed that Sev led to an increase in the proportion of multipolar neurons in the upIZ region and a significant decrease in the number of cells converted to bipolar morphology, accompanied by shorter length and incorrect orientation of leading processes. This process is closely related to the orientation of the Golgi apparatus [24]. We also further confirmed that the orientation of the Golgi apparatus appears to be an abnormal deviation, indicating that Sev inhibits the migration of neurons by affecting their normal morphological transition process. The cytoskeleton plays a crucial role in cell migration. Cofilin and profilin 1 are two proteins that regulate actin dynamics of the cytoskeleton [25]. Our results showed that Sev caused actin polymerization defects not only by affecting the expression of profilin 1 and the phosphorylation of cofilin, but also by affecting F‐actin remodeling. Neuronal migration and neurite growth are essential cellular events in neuronal development [26]. Neuronal somal translocation is determined by the neurite tension. To verify whether Sev inhibits neuronal migration through affecting the growth of neuronal neurites, we treated primary neurons with Sev in vitro. We found that Sev caused a significant reduction in neurite length and branch numbers. These data indicated that Sev inhibited neuronal migration by affecting neurite growth and neuronal development.

Iron is one of the essential trace elements for cell metabolism. Iron overload can cause cell damage and even apoptosis through oxidative stress [27]. One of the characteristics of ferroptosis is iron overload. Some recent studies demonstrated that Sev could induce ferroptosis [28, 29]. Our data suggested that Sev induced iron deposition in the embryonic mouse cortex, which was consistent with Sev inhibiting neuronal migration and axongenesis, indicating that iron metabolism dysfunction may be an important reason. To assess whether elevated iron levels inhibited neuronal migration, we treated mice with FAC and found that FAC inhibited neuronal migration, and its effect was significantly positively correlated with that of Sev‐treated mice. On the contrary, DFO as an iron chelator could significantly alleviate Sev‐induced neuronal migration defects and cortical dysplasia.

In summary, we demonstrated that Sev impaired neuronal migration and cortical development in mouse embryos by inhibiting the proliferation of NPCs and disrupting the dynamics of the actin cytoskeleton. Sev‐induced iron metabolism disorder may be one of the important mechanisms, and maintaining iron homeostasis may be an effective strategy for treating Sev‐induced cortical dysplasia.

## Author Contributions

Conceived and designed the experiments: Zhenhua Shi and Jianhua Zhang, Performed the experiments: Xincheng Li, Runjiao Cheng, Yong Zuo, Xiaoou Nie, and Jiaqi Wang; Analyzed the data: Xincheng Li, Liqiang Zhao, and Xiaopeng Liu; Wrote the paper: Zhenhua Shi, Jianhua Zhang, and Muhammad Naeem.

## Ethics Statement

The animal study was reviewed and approved by Hebei normal university ethics committee (NO. 2022LLSC033).

## Conflicts of Interest

The authors declare no conflicts of interest.

## Supporting information


Data S1.

## Data Availability

All the data can be obtained from the corresponding author.
