# Supplementary figures and images for: Sevoflurane Inhibits the Proliferation of Neural Precursor Cells and Neural Migration of Mice by Inducing Iron Metabolism Disorders
Source: CNS Neurosci Ther. 2025 Apr 9;31(4):e70369. doi: 10.1111/cns.70369 (PMC11979790; doi:10.1111/cns.70369)

Full unedited blot for Figure 3

Figure 3A

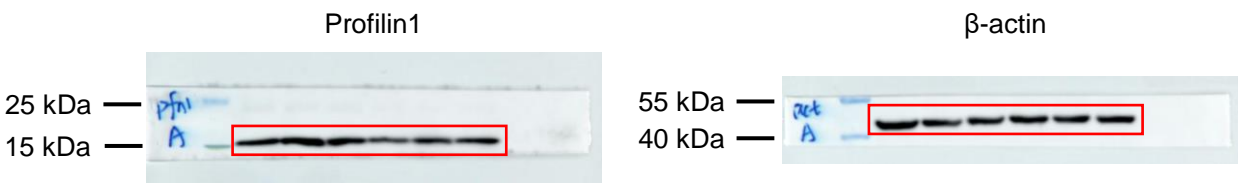

Figure 3C

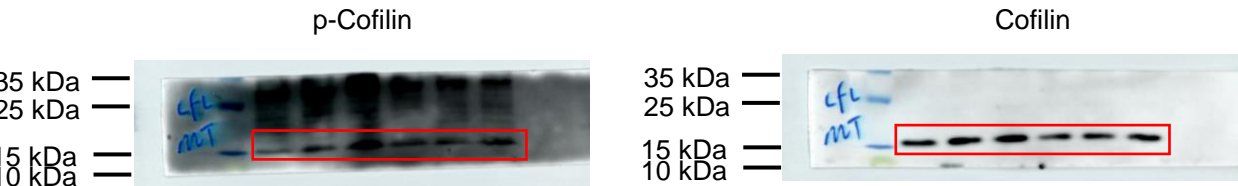

Figure 3E

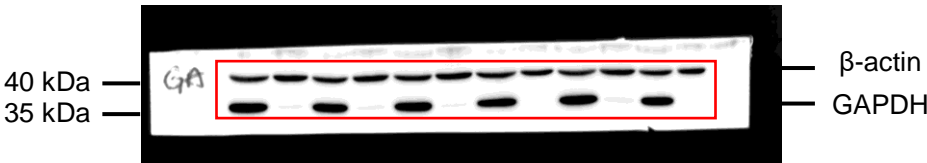

Figure 3G

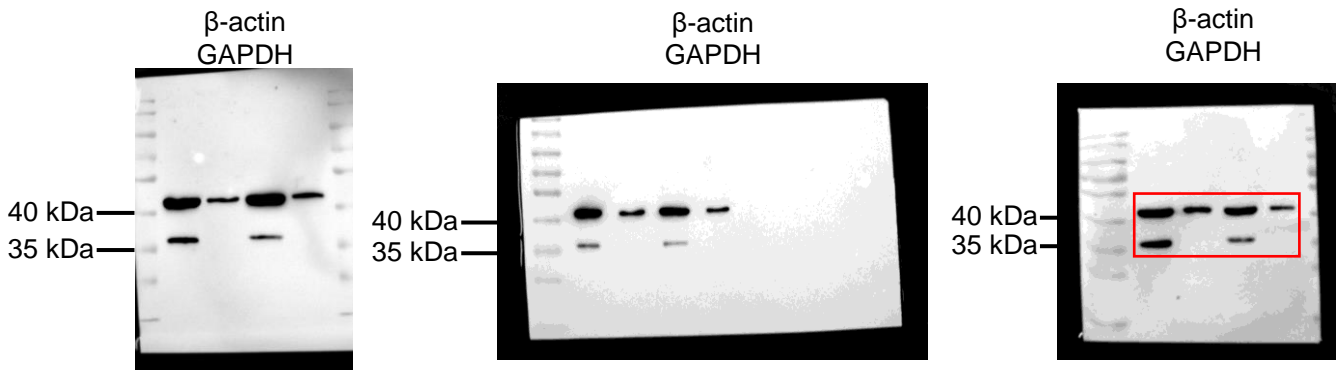

Figure 4A

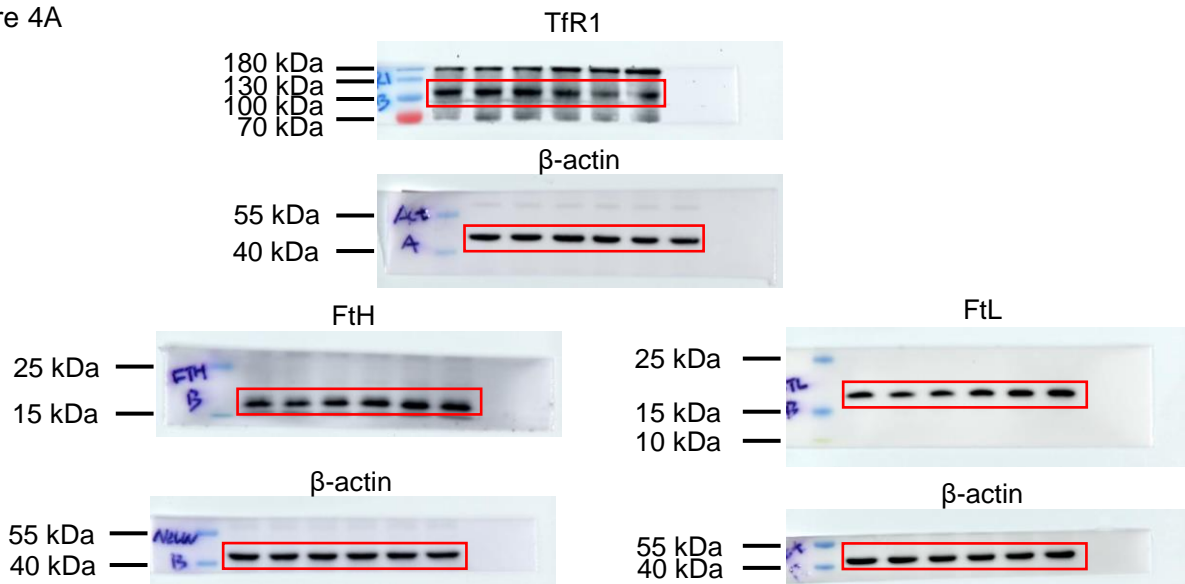

Figure 4E

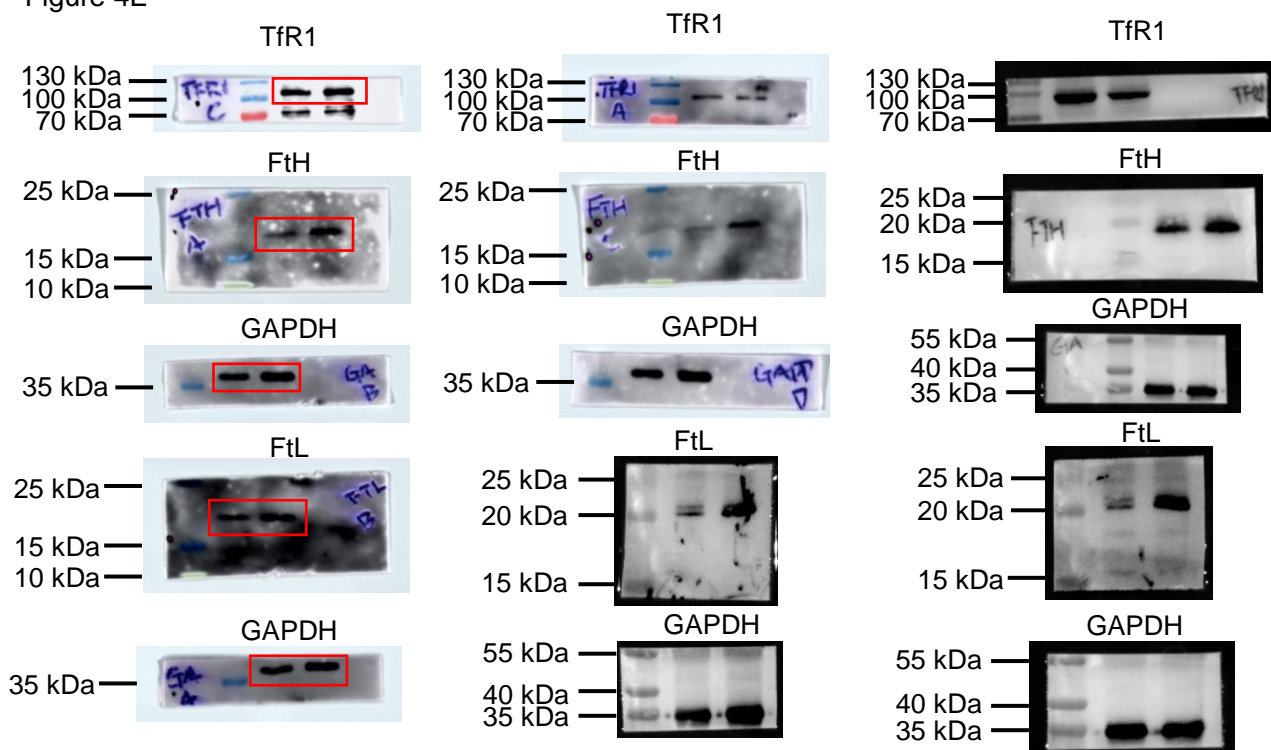

Figure 6A

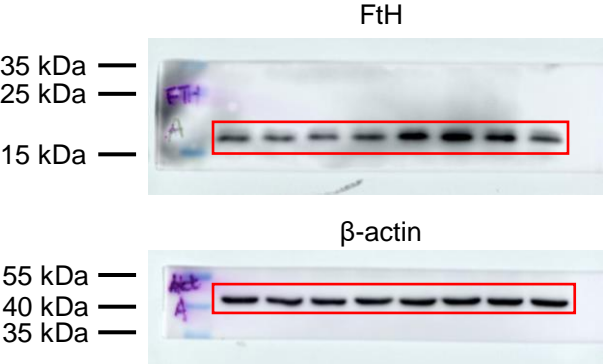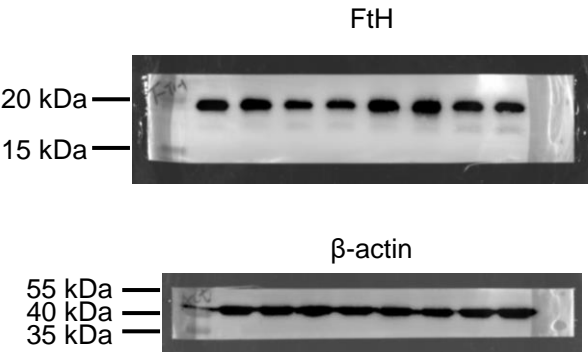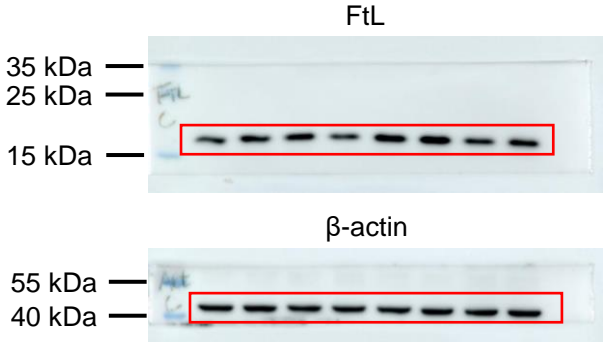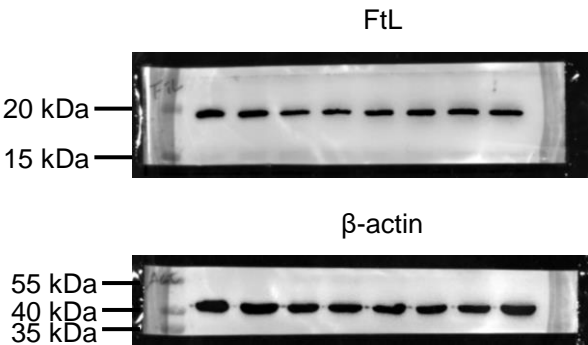

Supplement: Supplementary file 1 — Data S1. [file CNS-31-e70369-s001.pdf]
